# Integrating Virtual Mindfulness-Based Stress Reduction Into Inflammatory Bowel Disease Care: Mixed Methods Feasibility Trial

**DOI:** 10.2196/53550

**Published:** 2024-05-06

**Authors:** Kaitlyn Delaney Chappell, Diana Meakins, Melanie Marsh-Joyal, Allison Bihari, Karen J Goodman, Jean-Michel Le Melledo, Allen Lim, Farhad Peerani, Karen Ivy Kroeker

**Affiliations:** 1 Division of Gastroenterology Department of Medicine University of Alberta Edmonton, AB Canada; 2 Department of Psychiatry University of Alberta Edmonton, AB Canada; 3 Department of Psychiatry Royal Alexandra Hospital Edmonton, AB Canada

**Keywords:** inflammatory bowel disease, psychosocial care, multidisciplinary care, quality of care, quality of life, mental health, adult, adults, anxiety, depression, IBD, virtual mindfulness, feasibility trial, clinic, health facility, Canada, semistructured interview, psychiatrist, psychiatrists, videoconferencing, effectiveness, v-MBSR, coping, coping strategy

## Abstract

**Background:**

Individuals with inflammatory bowel disease (IBD) experience cycles of aggressive physical symptoms including abdominal pain, diarrhea, and fatigue. These acute symptoms regress and return, and chronic symptoms and complications often linger. The nature of the disease can also cause individuals to experience psychological distress including symptoms of anxiety and depression; however, unlike the physical symptoms of IBD, these psychological symptoms often remain untreated.

**Objective:**

This study aims to evaluate the feasibility, acceptability, and effectiveness of virtual mindfulness-based stress reduction (v-MBSR) for adults with IBD.

**Methods:**

IBD patients with self-reported anxiety or depression were recruited from clinics in Alberta, Canada to participate in an 8-week v-MSBR intervention. Eligible patients participated in v-MBSR delivered by psychiatrists using a videoconferencing platform. Primary feasibility outcomes included trial uptake, adherence, attendance, and attrition rates. Secondary effectiveness outcomes included measures of anxiety, depression, quality of life (QoL), and mindfulness. Effectiveness data were collected at 3 time points: baseline, at intervention completion, and 6 months after completion. To further assess feasibility and acceptability, participants were invited to participate in a semistructured interview after completing v-MBSR.

**Results:**

A total of 16 of the 64 (25%) referred patients agreed to participate in v-MBSR with the most common reason for decline being a lack of time while 7 of the 16 (43.8%) participants completed the program and experienced encouraging effects including decreased anxiety and depression symptoms and increased health-related QoL with both improvements persisting at 6-month follow-up. Participants described improved coping strategies and disease management techniques as benefits of v-MBSR.

**Conclusions:**

Patients with IBD were interested in a psychiatrist-led virtual anxiety management intervention, but results demonstrate v-MBSR may be too time intensive for some patients with IBD patients. v-MBSR was acceptable to those who completed the intervention, and improvements to anxiety, depression, and QoL were promising and sustainable. Future studies should attempt to characterize the patients with IBD who may benefit most from interventions like v-MBSR.

## Introduction

Inflammatory bowel diseases (IBDs), including Crohn disease (CD) and ulcerative colitis, are incurable diseases of the digestive tract with debilitating symptoms that can impair many aspects of a person’s life [1]. There is a large body of evidence suggesting the existence of a bidirectional connection between the gut and brain [2], thus research has begun to investigate the reciprocal relationship between mental health and the pathogenesis and severity of IBD [3,4]. Many high-quality studies have observed significant relationships between symptoms of psychological distress, including anxiety and depression, and both the severity of disease symptoms [5] as well as the development of flares [6].

Psychiatric comorbidities are common in people with IBD. A recent study reported that compared with the general population, adults with IBD have 10 times the risk of suicide, anxiety diagnoses, and depression diagnoses [7]. Further, a meta-analysis of over 30,000 patients with IBD estimated the prevalence of anxiety and depression symptoms in patients with active IBD to be 57.6% and 38.9%, respectively [8]. On top of these comorbid psychiatric risks, approximately 25%-33% of patients with IBD experience symptoms of psychological distress and posttraumatic stress symptoms from medical procedures, hospital stays, disease symptoms, and pain [9]. Despite these associations, most IBD patients’ mental health concerns often remain untreated [1,9].

Increasing one’s ability to cultivate mindfulness is a skill shown to have a protective effect on mental health by mediating the negative effects of the symptoms of psychological distress described above [10,11]. One of the aims of mindfulness-based interventions (MBIs), such as mindfulness-based stress reduction (MBSR), is to help develop the trait of mindfulness that can aid participants in reducing their feelings of anxiety and allow these benefits to persist for many years after completing the intervention. Specifically, MBSR encourages the development of mindfulness by teaching participants to approach their thoughts with openness and acceptance and by helping them to focus on and be nonjudgmental of the present moment [10,12,13]. Within the medical setting, in-person MBSR is effective in improving health-related quality of life (HRQoL) and reducing fatigue, stress, anxiety, and depression [14,15] in a variety of patient populations; however, a limited number of studies have focused on the IBD population. Further, there is little evidence to support the feasibility and effectiveness of the virtual delivery of mindfulness interventions, including virtual mindfulness-based stress reduction (v-MBSR).

The objective of this study was to assess if v-MBSR was feasible, acceptable, and effective for patients with IBD using both quantitative and qualitative approaches.

## Methods

### Design

This study was a multicenter, single-arm, feasibility trial. Study staff recruited patients with IBD from academic and nonacademic outpatient clinics in Edmonton, Alberta, Canada. We invited participants who completed the intervention to participate in a nested qualitative study.

### Ethical Considerations

The University of Alberta research ethics board granted ethics approval for the project as 2 separate studies (Pro00108955 and Pro00119852). Informed consent was obtained from all participants, and participants had the option to withdraw from the study at any time. All participant data were deidentified for analysis. Participants did not receive any compensation for their participation in the intervention or in the interviews.

### Participants and Recruitment

The research team recruited people with IBD experiencing stress, anxiety, or depression symptoms from 4 hospital outpatient clinics and a variety of gastroenterology clinics in Edmonton, Alberta, Canada between January and August 2022. Patients could self-refer using the posted study advertisements or could be referred by their gastroenterologist. Inclusion criteria included a confirmed diagnosis of CD or ulcerative colitis, age between 18 and 65 years, ability to communicate in English, and stable IBD and psychotropic medications for 6 weeks prior to the start of intervention. Exclusion criteria included ongoing corticosteroid use, having undergone surgery within 6 weeks of v-MBSR start date, and a history of or existing psychiatric-specific symptoms including psychotic or dissociative symptoms, severe active untreated drug use, severe self-injurious behavior, suicidal ideation, or cognitive impairment. We chose to add criteria regarding medication changes and surgery within 6 weeks of the start of the intervention in order to limit additional factors that could influence psychosocial well-being and quality of life (QoL).

At the time of recruitment, many gastroenterologists in Edmonton were conducting appointments via telehealth, so the study coordinator (KDC) contacted referred patients over the phone. If patients were not reached after 3 call attempts, they were categorized as “never reached”. Patients interested in participating signed an electronic consent form [16] and began the screening process. As research consent was only obtained after the referral, individuals who declined study participation were given the option to provide a reason for declining. The first step of screening involved participants completing an evaluation of their current anxious and depressive symptoms by completing a Patient-Health Questionnaire–Somatic, Anxiety, and Depressive Symptoms Scale (PHQ-SADS). Participants with at least mild symptoms (defined as a PHQ-SADS score ≥5) were eligible to proceed to the next step of screening—a 60-minute semistructured assessment with a psychiatrist. One of the collaborating psychiatrists conducted the assessment digitally, discussed the expectations within v-MBSR, and ensured the intervention was safe for the participant. After the assessment, eligible participants had 1 week to consider participating before confirming their preferred start date.

### Intervention

The MBSR intervention groups followed the standard protocol developed by Kabat-Zinn [10]. Participation involved 8 weekly sessions of 2.5 hours each, a 5-hour weekend session after week 6, and home practice lasting between 45 and 60 minutes per day. We provided participants with audio files and a program manual to assist in home practice. Phillips [17], a certified MBSR teacher, developed the protocol and audio recordings. The same 3 facilitators delivered the intervention to groups of 7-9 participants. All facilitators regularly deliver MBSR as part of their clinical practice and are qualified MBSR teachers. The facilitators delivered the weekly sessions in the evenings to accommodate participants attending school or work during the daytime, and the videoconferencing platform met legislative and provincial safety and privacy guidelines.

### Feasibility and Acceptability Outcome Measures

The primary aim of this study was to assess the feasibility and acceptability of v-MBSR in adults with IBD. We selected 4 primary outcomes, adapted from the framework and recommendations for feasibility trials [18,19] to assess feasibility: recruitment success, adherence, attendance, and attrition. We defined recruitment success verbally as trial uptake, and mathematically as the number of participants who enrolled divided by the total number of participants referred. We assessed adherence in the participants who completed the intervention. We defined adherence as the average number of minutes that participants who completed the intervention practiced per day divided by the average number of minutes they were asked to practice per day. As per the protocol of MBSR [10], the recommended home practice is 45 minutes each day. We assessed attendance and attrition together and verbally defined this combined measure as the number of participants who completed the intervention. As with adherence, we defined completion as recommended by the original MBSR protocol [10]: participants were considered to have completed v-MBSR if they attended 6 out of 8 weekly sessions as well as the weekend session. To assess this mathematically, we calculated the proportion of participants who we considered to have completed the intervention by dividing the number of enrolled participants who did not discontinue the intervention and met the defined completion criteria by the total number of participants who enrolled.

### Short-Term Effectiveness Outcome Measures

The secondary outcomes of the trial were the short-term effectiveness of v-MBSR in the participants who completed it across the domain of psychosocial functioning. We defined the intervention effect as a change from baseline in psychosocial functioning following the intervention, with participants acting as their own controls. We also collected measures of disease activity to evaluate if patients experienced active disease during or after their participation in the group. The collection of short-term effectiveness data occurred at 3 time points: pregroup, postgroup, and 6-month postgroup. We collected baseline or pregroup data up to 2 weeks before the v-MBSR group started, postgroup data at 1 week following the completion of the group, and 6-month follow-up data 24-26 weeks after participants completed the group.

### Psychosocial Functioning Outcome Measures

#### Psychological Distress (Anxiety and Depression Symptoms)

We measured psychological distress using assessment tools for somatic, anxiety, and depressive symptoms; HRQoL; dispositional mindfulness; and self-compassion.

The PHQ-SADS [20] is a 3-part scale used to assess anxiety, depression, somatization, and general stress symptoms. Scores range from 0 to 27 with higher scores indicating a more severe symptom burden. PHQ-SADS above 5 indicates the presence of at least mild symptoms and a score above 10 is considered clinically concerning [20].

#### Health-Related Quality of Life (HRQoL)

The Short Inflammatory Bowel Disease Questionnaire (SIBDQ) [21] is a tool used to assess disease-specific HRQoL in people with IBD. The 10-item questionnaire is adapted from the Inflammatory Bowel Disease Questionnaire and assesses QoL across 4 dimensions: bowel, systemic, social, and emotional. Scores range from 10 to 70 with lower scores indicating poorer QoL. Use of the SIBDQ, authored by Dr Jan Irvine et al, was made under license from McMaster University, Hamilton, Canada.

#### Mindfulness

The Mindful Attention and Awareness Scale [22] is a tool used to assess dispositional mindfulness, also referred to as the trait of mindfulness. The 15-item questionnaire focuses on respondents’ open and receptive awareness and their ability to focus their attention on the present. Scores range from 1 to 6 and higher scores indicate greater levels of dispositional mindfulness.

#### Self-Compassion

The Short Form Self-Compassion Scale [23] is a scale that assesses respondents’ ability to be kind and understanding to themselves in instances of pain and failure. The 12-item scale is adapted from the Self-Compassion Scale. Scores range from 1 to 5 with higher scores indicating greater levels of self-compassion.

### Disease Activity Outcome Measures

The Harvey Bradshaw Index [24] and the Partial Mayo Score [25] were used to assess IBD clinical symptoms and disease activity. These clinical indicators of disease were supplemented with inflammatory markers of disease activity, including C-reactive protein and fecal calprotectin (FCP) if laboratory results were available within 2 months of the time point of interest.

### Baseline Characteristics

We collected participant baseline and demographic information 0-2 weeks before the group start date. Information collected included age, self-identified gender, type of IBD, age at diagnosis, employment status, a list of medications, and the adverse childhood experience (ACE) questionnaire.

The ACE Scale [26] is a 10-item questionnaire that assesses childhood rearing and maltreatment contexts with scores ranging from 0 to 10. Previous studies have associated higher ACE scores with mental illness indicators including emotional distress and poorer general health outcomes [27]. ACE scores can be useful to identify patients who may have greater mental health needs now or in the future.

### Statistical Analysis

We included all participants with baseline data in the pregroup analysis. Only participants who met the completion criteria were included in the postgroup and 6-month follow-up analysis. We performed the calculations of descriptive statistics and analyses using Stata (version 17.0; StataCorp) [28]. Our calculations included proportions as well as means with 95% CIs to demonstrate precision. We compared the participants’ preintervention and postintervention mean outcome scores with the preintervention score acting as the control.

### Nested Qualitative Study

To understand participants’ experiences with v-MBSR, we invited participants who completed the intervention to participate in a semistructured interview. The interviews aimed to gather more data regarding the feasibility, acceptability, and effectiveness of v-MBSR and to identify contextual factors that may be relevant to implementing a mental health intervention such as v-MBSR in IBD care.

We designed the interviews from the philosophical perspective of interpretive description as described by Thorne et al [29,30]. The interpretive description focuses on gaining a deeper understanding of the participant’s experience and because of this, reaching saturation is not a priority of this type of inquiry. We invited all participants who completed v-MBSR to participate in the interviews. All videoconference interviews took place between November and December 2022. Subsequently, the first author (KDC) transcribed the video interviews and generated codes and then themes from the transcripts using latent thematic analysis. In keeping with best practices for qualitative inquiry, a critical friend with experience in qualitative methods (AB) evaluated and audited the data.

## Results

### Baseline Characteristics

Table 1 summarizes the characteristics of participants who enrolled in the feasibility trial. The average age was 36 (range 18-55) years and 62.5% (n=10) of participants were female, 50% (n=8) of participants had CD, and 68.8% (n=11) of the participants were employed or studying full-time. At the time of their participation, 87.5% (n=14) of participants were using a biologic to treat their disease and 56.3% (n=9) of participants were using at least 1 psychotropic medication with the most common (8/16, 50%) being antidepressants. The average ACE score was 3.0 (95% CI 1.6-4.5).

**Table 1:** Participant demographic information including self-identified sex, age, diagnosis information, education, and medication details (N=16).

Characteristics		Values, n (%)
**Sex**		
	Female	10 (62.5)
	Male	6 (37.5)
**Type of IBD^a^**		
	CD^b^	8 (50)
	UC^c^	8 (50)
**Age (years)**		
	18-24	3 (18.8)
	25-34	3 (18.8)
	35-44	6 (37.5)
	45-54	3 (18.8)
	55-65	1 (6.3)
**Age at diagnosis (years)**		
	<18	4 (25)
	18-29	7 (43.8)
	>30	5 (31.3)
**Years since diagnosis**		
	<5	5 (31.3)
	5-10	5 (31.3)
	>10	6 (37.5)
**Employment status**		
	Employed full-time	7 (43.8)
	Studying full-time	4 (25)
	Unemployed/retired	2 (12.5)
	Employed part-time	1 (6.3)
	Self-employed	1 (6.3)
	Other	1 (6.3)
**IBD medication**		
	Biologic therapy only	9 (56.3)
	Combination therapy (biologic and immunosuppressant)	4 (25)
	5-ASA^d^ only	1 (6.3)
	Biologic and 5-ASA	1 (6.3)
	None	1 (6.3)
**Psychotropic medication**		
	No	7 (43.8)
	Yes	9 (56.3)
**Amount of psychotropic medications given to people who opted “Yes”**		
	1	3 (33.3)
	2	2 (22.2)
	3+	5 (55.6)
**Psychotropic medication by type**		
	Antidepressant	8 (50)
	Sedative/hypnotic	5 (31.3)
	Mood stabilizer/anticonvulsant	2 (12.5)
	Antipsychotic	2 (12.5)
	Other	2 (12.5)
	Stimulant	1 (6.3)

^a^IBD: inflammatory bowel disease.

^b^CD: Crohn disease.

^c^UC: ulcerative colitis.

^d^5-ASA: 5-aminosalicylic acid.

### Feasibility and Acceptability Outcomes

#### Recruitment

During the 8-month recruitment period, 64 patients were referred to the trial. Nearly all patients (60/64, 93.8%) were referred by their gastroenterologist with 50% (n=32) of referrals coming from a gastroenterologist working at an academic center and 76.6% (n=49) of referrals being from male gastroenterologists. Overall, 7 individual gastroenterologists referred patients to the study.

**Figure 1:**
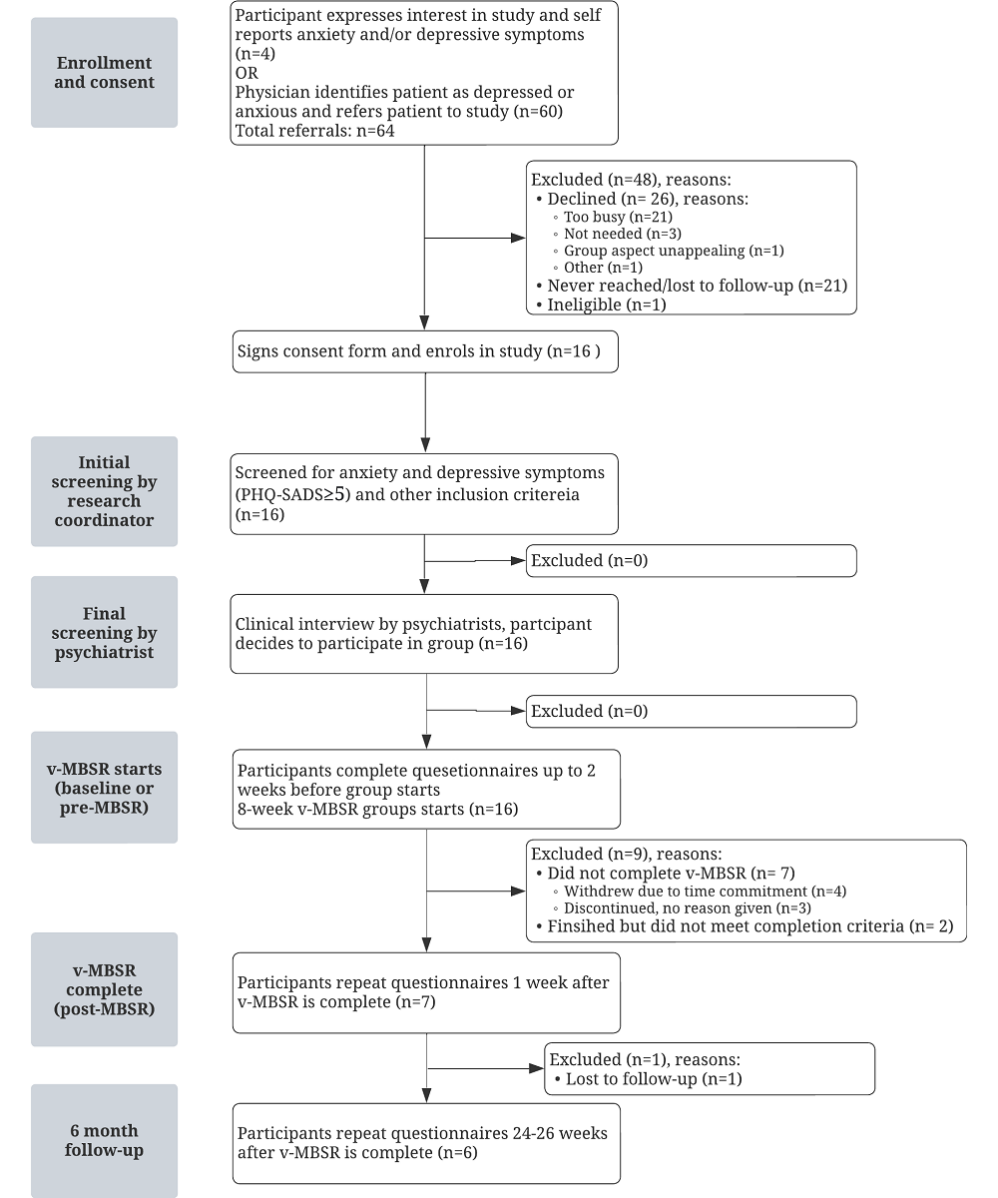
Flow diagram of participant progress through all stages of the study reasons for exclusion noted at each stage. MBSR: mindfulness-based stress reduction; PHQ-SADS: Patient-Health Questionnaire–Somatic, Anxiety, and Depressive Symptoms Scale; v-MBSR: virtual mindfulness-based stress reduction.

Of the 64 patients referred, 25% (n=16) of patients enrolled in 1 of the 2 offered v-MBSR groups. Patient flow through the trial is shown in Figure 1. Other feasibility data including reasons for declining, attendance, adherence, and attrition are included in Table 2. Of note, the participants’ average adherence rate corresponded to 48% (21.7 of 45 minutes) of the recommended daily practice time. Further, no patients were excluded due to low PHQ-SADS scores or following the completion of the psychiatric assessment and all 7 participants who discontinued did so before the third weekly session.

**Table 2:** Recruitment, attrition, attendance, and adherence results.

Characteristics		Values
**Recruitment (n=64), n (%)**		
	Enrolled	16 (25)
	Declined	26 (40.6)
	Lost to follow-up/never reached	21 (32.8)
	Ineligible	1 (1.56)
**Reasons for declining (n=26), n (%)**		
	Too busy/lack of time	21 (80.8)
	Help not necessary	3 (11.5)
	Group aspect unappealing	1 (3.85)
	Not enough help	1 (3.85)
**Attrition and attendance (n=16), n (%)**		
	Completed	7 (43.8)
	Finished, but did not complete	2 (12.5)
	Discontinued	7 (43.8)
**Reasons for discontinuing (n=7), n (%)**		
	Lack of time	4 (57.1)
	No reason noted	3 (42.8)
**Adherence (n=6; minutes), mean (95% CI)**		
	Average practice per day	21.7 (13.1-30.2)

#### Participant Interviews: Barriers to v-MBSR

**Figure 2:**
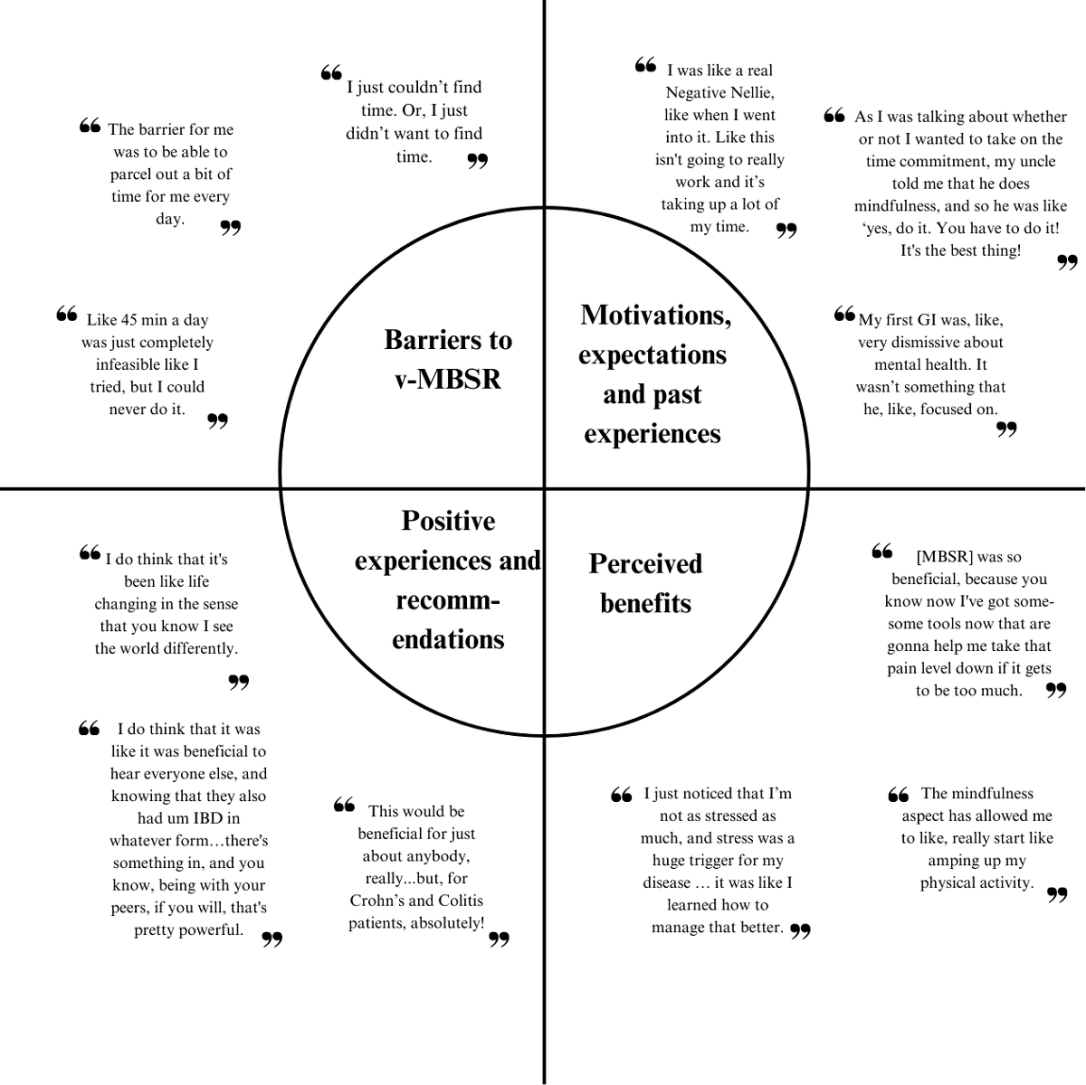
Participant quotes from interviews organized by the 4 primary topics of discussion: barriers, motivations, experiences, and benefits. IBD: inflammatory bowel disease; MBSR: mindfulness-based stress reduction; v-MBSR: virtual mindfulness-based stress reduction.

We assessed barriers to the intervention in the nested qualitative study. When we asked participants to identify challenges to participating in the intervention, excessive time related to home practice was mentioned as the largest barrier by all 5 participants. One participant noted that the inflexibility of the weekly session (ie, having to attend the weekly session on the same day and at the same time every week) was also challenging. Quotes from interviews are presented in Figure 2.

### Characterizing Those Who Completed the Intervention

We compared participants who completed the intervention to the remaining participants across a variety of characteristics including referral details, demographics, and baseline scores, as summarized in Tables 3 and 4. Compared with participants who did not complete v-MBSR, those who completed the intervention were all female and more likely to be older, receive care from a gastroenterologist at a nonacademic center, have CD, work or study full-time, and have higher average ACE scores. Notably, the group of participants who completed the intervention had higher average ACE scores despite having comparable psychosocial functioning scores at baseline.

**Table 3:** Baseline demographics and characteristics of participants organized by participant completion status.

		All participants (n=16)	Complete participants (n=7)	Incomplete participants (n=9)
**Participant sex, n (%)**				
	Female	10 (62.5)	7 (100)	3 (33.3)
**Employment status, n (%)**				
	Full-time work/study	11 (68.8)	4 (57.1)	7 (77.8)
**Taking psychotropics, n (%)**				
	Yes	9 (56.3)	4 (57.1)	5 (55.6)
**Disease type, n (%)**				
	CD^a^	8 (50)	5 (71.4)	3 (33.3)
Age (years), mean (95% CI)		36.4 (30.6-42.2)	38.1 (27.3-49.0)	35.0 (26.7-43.3)
Years since diagnosis, mean (95% CI)		10.4 (5.6-15.1)	9.7 (0.0-21.0)^b^	10.9 (6.2-15.6)
ACE^c^ score, mean (95% CI)		3.06 (1.61-4.51)	3.43 (0.71-6.14)	2.78 (0.71-4.84)
**Baseline scores,** **mean** **(95% CI)**				
	PHQ-SADS^d^	11.8 (9.2-14.3)	11.9 (7.5-16.3)	11.7 (7.8-15.6)
	SIBDQ^e^	41.0 (35.7-46.2)	39.6 (29.0-50.2)	42.0 (35.0-49.0)
	MAAS^f^	4.0 (3.5-4.6)	4.3 (3.3-5.3)	3.9 (3.0-4.8)
	SCS^g^	2.3 (2.0-2.6)	2.2 (1.6-2.8)	2.2 (1.9-2.5)

^a^CD: Crohn disease.

^b^95% CI imprecise due to outliers, range 3-36.

^c^ACE: adverse childhood experience.

^d^PHQ-SADS: Patient-Health Questionnaire–Somatic, Anxiety, and Depressive Symptoms Scale.

^e^SIBDQ: Short Inflammatory Bowel Disease Questionnaire.

^f^MAAS: Mindful Attention and Awareness Scale.

^g^SCS: Self-Compassion Scale.

**Table 4:** Characteristics of referring physicians organized by participant completion status.

Characteristics		All participants (n=16), n (%)	Complete participants (n=7), n (%)	Incomplete participants (n=9), n (%)
**Location of referring physician**				
	Academic center	9 (56.3)	2 (28.6)	7 (77.8)
**Sex of referring physician**				
	Female	3 (18.8)	1 (14.3)	2 (22.2)

### Participant Interviews: Motivations, Expectations, and Past Experiences

We asked interview participants, all of whom completed v-MBSR, about their motivations to enroll in v-MBSR. All 5 noted they were motivated to engage in v-MBSR by at least 1 of the following factors: previous experience with mindfulness, a desire to improve their QoL and disease management through learning, their own research, specific intervention features such as the intervention sessions being led by a professional, and recommendations from others including family, friends, or health care providers. The 3 most common motivations were personal research, the desire to improve through learning, and recommendations from others.

We also asked interview participants to recall their expectations of v-MBSR before they participated in the intervention. Four of 5 interview participants had limited or negative expectations of the intervention. Specifically, these expectations included feelings of apprehension regarding their enjoyment of and success with the intervention as well as strong negative feelings regarding the length of the weekly meeting.

Additionally, we asked interview participants about their previous experience with trying to improve their well-being. All 5 participants described previously engaging in activities to improve their physical well-being including diet and exercise; 4 of 5 participants had tried mental health improvements including finding supportive social circles and seeing a mental health care practitioner such as a therapist or psychiatrist.

### Participant Interviews: Positive Experiences and Recommendations

All 5 interview participants described their experience in v-MBSR positively and indicated that being able to participate in the group with other people with IBD contributed to their positive experience. Two of 5 participants also added that their participation led to changes in their attitudes and perceptions which contributed to the positive experience.

All 5 participants said they would recommend v-MBSR to others, and 3 participants emphasized that they would continue to practice mindfulness going forward and share their experience and tools with others.

### Short-Term Effectiveness Outcomes

All 16 enrolled participants provided baseline data before the intervention began. Those who completed the intervention also provided data immediately after and 6 months after the intervention ended. One of the 7 participants who completed the intervention was lost to follow-up immediately after the intervention and did not provide postintervention data.

### Psychosocial Functioning Outcomes

**Figure 3:**
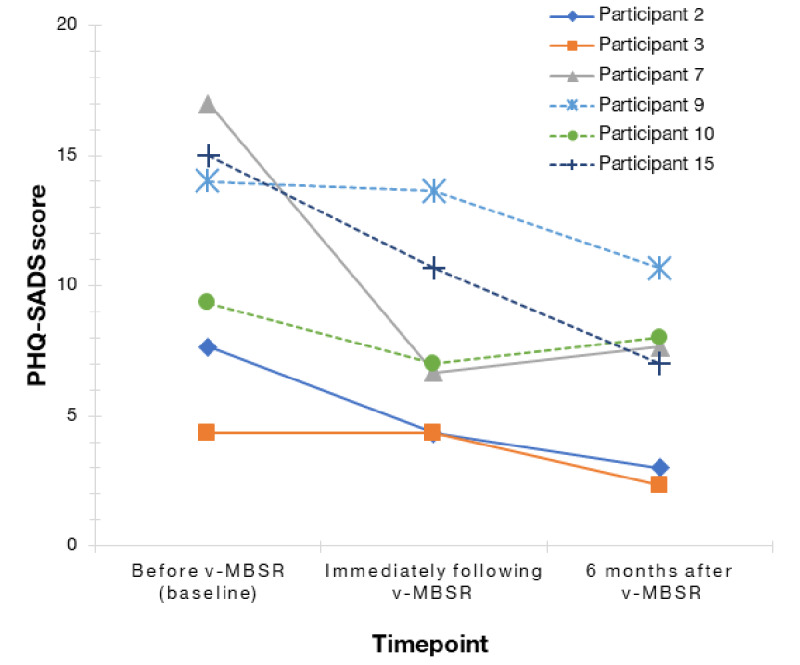
Participant anxiety scores at baseline, postintervention, and 6 months postintervention organized by participant. PHQ-SADS: Patient-Health Questionnaire–Somatic, Anxiety, and Depressive Symptoms Scale; v-MBSR: virtual mindfulness-based stress reduction.

**Figure 4:**
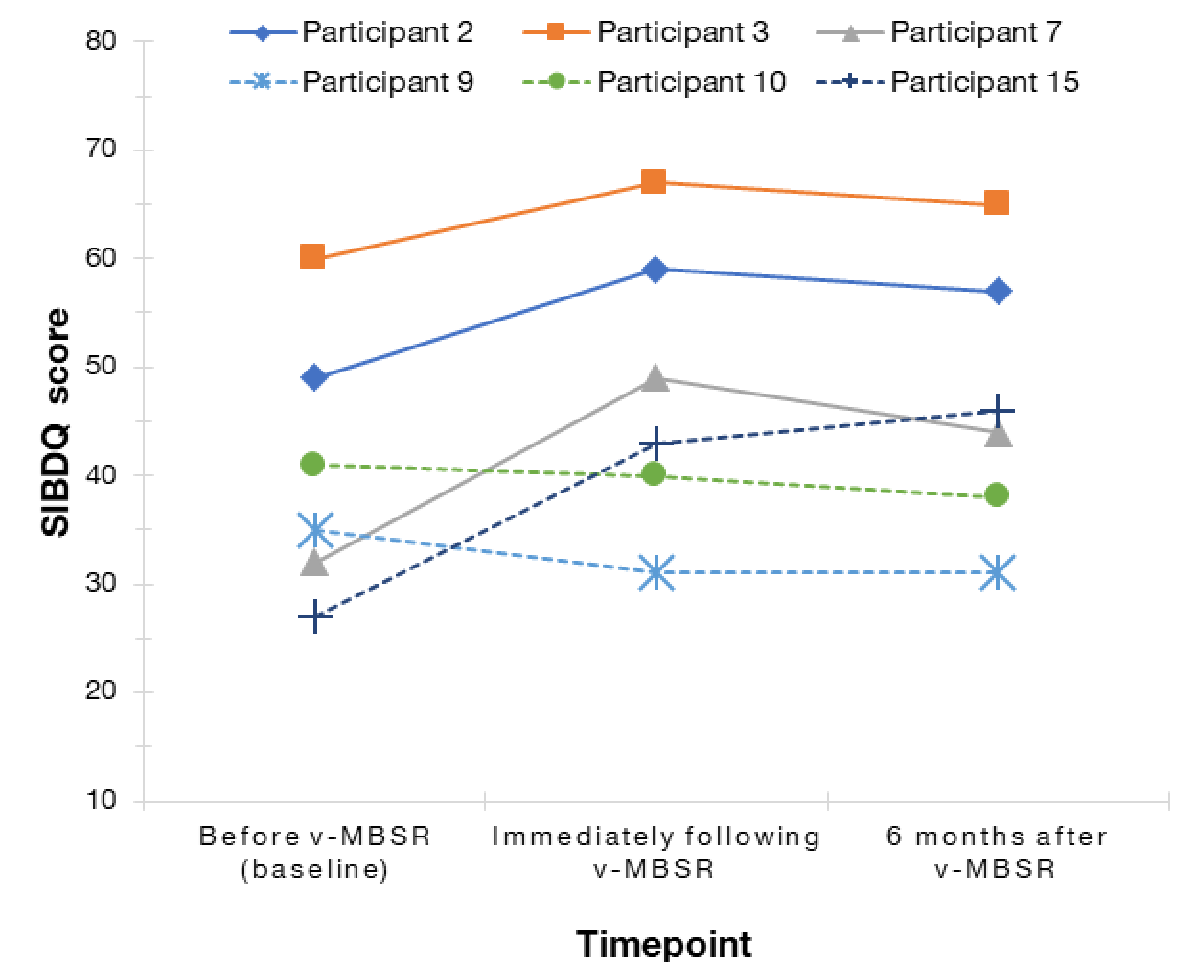
Participant quality of life scores baseline, postintervention, and 6 months postintervention organized by participant. SIBDQ: Short Inflammatory Bowel Disease Questionnaire; v-MBSR: virtual mindfulness-based stress reduction.

Figure 3, Figure 4, and Table 5 present psychosocial outcomes at all 3 time points, with results for individual participants as well as means at each time point. Participants’ mean PHQ-SADS score decreased from 11.2 at baseline to 7.8 immediately following the intervention and further to 6.4 at the 6-month follow-up. Participants’ SIBDQ scores showed a similar improvement. Their mean SIBDQ score increased from 40.7 at baseline to 48.1 postintervention. Participants’ mean SIBDQ score was 46.8 at the time of 6-month follow-up demonstrating sustained improvement.

**Table 5:** Participant psychosocial outcome scores at all 3 time points organized by participant.

Participant ID	Completion status	PHQ-SADS^a^			SIBDQ^b^				MAAS^c^				SCS-SF^d^		
		Pre^e^	Post^f^	6M^g^	Pre	Post	6M	Pre		Post	6M	Pre		Post	6M
1	No^h^	17.3	N/A^i^	N/A	33	N/A	N/A	3.1		N/A	N/A	1.8		N/A	N/A
2	Yes	7.7	4.3	3.0	49	59	57	5.1		4.3	4.1	2.0		3.1	2.7
3	Yes	4.3	4.3	2.3	60	67	65	5.6		4.5	4.8	2.8		3.2	2.9
4	No	12.3	N/A	N/A	44	N/A	N/A	2.2		N/A	N/A	1.9		N/A	N/A
5	No	10.0	N/A	N/A	46	N/A	N/A	3.3		N/A	N/A	1.9		N/A	N/A
6	No	12.7	N/A	N/A	52	N/A	N/A	5.5		N/A	N/A	2.8		N/A	N/A
7	Yes	17.0	6.7	7.7	32	49	44	3.1		3.0	3.9	1.8		2.0	2.5
8	Yes^j^	15.7	N/A	N/A	33	N/A	N/A	3.5		N/A	N/A	3.3		N/A	N/A
9	Yes	14.0	13.7	10.7	35	31	31	4.3		3.4	3.9	3.0		3.0	2.6
10	Yes	9.3	7.0	8.0	41	40	38	4.1		3.2	2.3	1.8		3.1	1.6
11	No	21.3	N/A	N/A	28	N/A	N/A	2.8		N/A	N/A	2.1		N/A	N/A
12	No	10.0	N/A	N/A	47	N/A	N/A	4.3		N/A	N/A	2.7		N/A	N/A
13	No^h^	10.3	N/A	N/A	30	N/A	N/A	4.3		N/A	N/A	2.2		N/A	N/A
14	No	4.7	N/A	N/A	50	N/A	N/A	3.7		N/A	N/A	2.0		N/A	N/A
15	Yes	15.0	10.7	7.0	27	43	46	3.7		3.3	3.3	1.8		3.8	3.5
16	No	6.7	N/A	N/A	48	N/A	N/A	5.6		N/A	N/A	2.7		N/A	N/A
N/A	Mean in all participants	11.8	N/A	N/A	41	N/A	N/A	4.0		N/A	N/A	2.3		N/A	N/A
N/A	Mean in completed participants only (95% CI)	11.9 (6.1-16.3)	7.8 (3.9-11.7)	6.4 (3.1-9.8)	40 (28-53)	48 (34-62)	47 (34-60)	4.3 (3.3-5.3)		3.6 (3.0-4.2)	3.7 (2.8-4.6)	2.2 (1.6-2.8)		3.0 (2.4-3.6)	2.6 (2.0-3.3)

^a^PHQ-SADS: Patient-Health Questionnaire–Somatic, Anxiety, and Depressive Symptoms Scale.

^b^SIBDQ: Short Inflammatory Bowel Disease Questionnaire.

^c^MAAS: Mindful Attention and Awareness Scale.

^d^SCS-SF: Short Form Self-Compassion Scale.

^e^Pre: score preintervention.

^f^Post: score postintervention.

^g^6M: score 6 months postintervention.

^h^Did not complete, but did finish.

^i^N/A: not applicable.

^j^Lost to follow-up.

### Participant Interviews: Perceived Benefits

When asked about the benefits of the program, all 5 interview participants noted the main benefits arose from the new techniques they were able to develop. All 5 indicated that they developed coping skills allowing them to better manage their anxiety and stress in addition to new focus and mindfulness tools. Four of 5 participants also noted they benefited from novel techniques that allowed them to manage disease symptoms and pain.

### Disease Activity Outcomes

As expected, given the study eligibility criteria, all participants started the intervention in inflammatory remission. One participant (Participant #15) experienced a flare after the intervention concluded, indicated by FCP levels greater than 250 µg/mg. All other participants remained in clinical remission throughout and in the 6 months following the intervention.

## Discussion

### Principal Results

Together, the mix of quantitative and qualitative data from this study provides insight into the feasibility, acceptability, and preliminary effectiveness of v-MBSR for patients with IBD. To our knowledge, this is the first study to evaluate v-MBSR in this patient population and also the first study to use qualitative methods to explore experiences with MSBR among IBD patients.

Two other studies examined MBSR as a treatment for people with IBD: a 2014 randomized controlled trial conducted by Jedel et al [31] and a 2016 controlled trial conducted by Neilson et al [32]. Of note, both of these trials delivered MBSR in-person. Despite participants in our study having similar personal and baseline health characteristics to the participants of both the Jedel et al [31] and the Neilson et al [32] trials, our study had lower uptake and substantially higher attrition. This difference is surprising, especially considering the potential benefits of telehealth. Several high-quality studies have identified travel distance as one of the largest barriers to accessing health care and one of the largest reasons for discontinuing treatment [33,34], so it is puzzling that adapting MBSR to be delivered digitally via telehealth led to lower uptake and higher attrition rates compared with MBSR delivered in-person. Moreover, a previous study of MBSR in the general population demonstrated similar attrition when comparing in-person delivery of MBSR with digital delivery [35]. In view of all 3 studies being conducted in different geographical locations and at different times, it is possible that the study populations differ in their comfort levels with telehealth; however, a recent survey of IBD patients in Alberta found that over 80% of respondents were comfortable using virtual care options and agreed they were satisfied with telehealth as a treatment modality [36], and a previous study in the same geographical area with a similar IBD population found no challenges with attrition [37]. While attrition observed in our study could reflect random variation arising from the small number of participants, future studies should investigate if v-MBSR groups are noninferior to in-person MBSR groups with respect to completion.

In addition to the surprisingly high attrition, it is curious that only women completed the intervention in our trial, given that this difference was not reported in either of the previous MBSR for IBD trials [31,32] or in other trials of MBIs for IBD [37,38]. In contrast to the above studies, authors of a recent systematic review [39] of adherence predictors for digital psychological interventions reported that female sex was a predictor of increased adherence to psychological interventions. Further, several reviews of mindfulness in general and patient populations have found that females are more likely than males to practice meditation to gravitate toward MBIs and to report greater improvements in symptoms of psychological distress from MBIs [40,41]. Future studies may consider investigating how gender and sex may impact enrollment and attrition in MBIs.

Multiple systematic reviews have found MBIs, including MBSR, to be effective tools to reduce anxiety and improve QoL in people with IBD [42,43]. The participants that completed v-MBSR in our study noted very similar effects that persisted even 6 months after the completion of the intervention, during which time no additional instruction or resources were provided. Despite the limitations of our small study, the magnitude and sustainability of improvements in psychosocial functioning outcomes reported by participants are promising. The interviews also provided rich data regarding the effectiveness of the program. Despite 4 of the 5 interview participants noting negative or limited expectations before beginning the intervention, all 5 reported positive experiences with v-MBSR, highlighting its nonspecific benefits. The interview participants also elaborated on specific benefits of v-MBSR including increased control coping with anxiety, managing their disease, and focusing. This is particularly important because having a sense of control over the physical and mental health symptoms of IBD is paramount for patients, and losing this sense of control often leads to feelings of anxiety, depression, and distress [44].

### Limitations

This study is not without limitations, with the largest being the small sample size. Because of this limitation, our analysis lacked sufficient data for statistically precise estimates of differences between groups. Sufficient statistical precision could have allowed us to identify characteristics associated with completing and benefiting from the intervention. Only women successfully completed our intervention, so we were unable to describe the effects of the intervention on men. While this limits the generalizability of our results, this difference can be used to inform recruitment in similar studies in the future. For example, it may be helpful to offer male participants more support in future studies of MBIs in order to increase their ability and desire to finish the intervention.

Our effectiveness data may further be limited because the outcomes were measured with self-administered questionnaires, which can be subject to social desirability bias and personal interpretation. In contrast to this limitation, our questionnaires were advantageous since they captured improvements to patient-reported outcomes such as HRQoL, which are neglected by objective measures of disease such as FCP. Our interviews were limited to participants who had completed the intervention, but they still provided some beneficial information for future recruitment for MBIs in this population. For instance, the results of our interviews suggest patients with previous mindfulness experience may be a desirable target for MBSR, as this was a motivating factor for participation identified during several of the interviews.

### Conclusions

Despite some of the struggles with recruitment and retention, there was substantial interest from patients with IBD and providers in a free, virtual, and psychiatrist-led stress reduction intervention, and participants who completed the intervention saw significant and sustained improvements to their psychosocial health. In view of these favorable results, v-MBSR may serve as a promising integrated treatment for a subset of patients with IBD who are able and willing to manage the intervention’s large time commitment and could be a beneficial tool to help patients with IBD regain a sense of control over their physical and mental health while they are in remission. Future studies should investigate if telehealth-based group interventions are noninferior to their face-to-face equivalents and characterize the patients with IBD who are able to commit to and benefit from intensive MBIs.

## Abbreviations

ACE: adverse childhood experience
CD: Crohn disease
FCP: fecal calprotectin
HRQoL: health-related quality of life
IBD: inflammatory bowel disease
MBI: mindfulness-based intervention
MBSR: mindfulness-based stress reduction
PHQ-SADS: Patient-Health Questionnaire–Somatic, Anxiety, and Depressive Symptoms Scale
QoL: quality of life
SIBDQ: Short Inflammatory Bowel Disease Questionnaire
v-MBSR: virtual mindfulness-based stress reduction

## Data Availability

All data generated during this study are available in this published thesis (KDC) [45].
